# Vascular function in the aging human brain during muscle exertion

**DOI:** 10.18632/aging.204052

**Published:** 2022-05-02

**Authors:** Maijian Zhu, Tania Xu Yar Lee, Yu-Wen Hsieh, Li-Fan Lai, Giancarlo Condello, Cyril J. Donnelly, Marc Smith, Sareena Hanim Hamzah, Boon-Hooi Lim, Chih-Yang Huang, Nai-Fang Chi, Chia-Hua Kuo

**Affiliations:** 1 Laboratory of Exercise Biochemistry, University of Taipei, Taipei City 11153, Taiwan, ROC; 2 Department of Medicine and Surgery, University of Parma, Via Gramsci, Parma 43126, Italy; 3 Rehabilitation Research Institute of Singapore, Nanyang Technological University, Singapore; 4 Body Composition Technologies, Pty Ltd., South Perth, Western Australia, Australia; 5 Centre for Sport and Exercise Sciences, University Malaya, Kuala Lumpur 50603, Malaysia; 6 Cardiovascular and Mitochondrial Related Disease Research Center, Hualien Tzu Chi Hospital, Buddhist Tzu Chi Medical Foundation, Hualien 970, Taiwan, ROC; 7 Center of General Education, Buddhist Tzu Chi Medical Foundation, Tzu Chi University of Science and Technology, Hualien 970, Taiwan, ROC; 8 Department of Medical Research, China Medical University Hospital, China Medical University, Taichung 404, Taiwan, ROC; 9 Graduate Institute of Biomedical Sciences, China Medical University, Taichung 404, Taiwan, ROC; 10 Department of Neurology, National Yang Ming Chiao Tung University School of Medicine, Taiwan, ROC; 11 Department of Neurology, Taipei Veterans General Hospital, Taiwan, ROC

**Keywords:** frailty, vascular function, muscle strength, endothelial function, NIRS

## Abstract

To determine how brain oxygenation is stably maintained during advancing age, cerebral oxygenation and hemoglobin were measured real-time at 10 Hz using near-infrared spectroscopy (NIRS) at rest (30 seconds) and during a 10-repeated handgrip strength test (30 seconds) for 834 adults (M/F = 45/55%) aged 20–88 y. The amplitude of cerebral hemodynamic fluctuation was reflected by converting 300 values of % oxygen saturation and hemoglobin of each 30-second phase to standard deviation as indicatives of brain oxygenation variability (BOV) and brain hemodynamic variability (BHV) for each participant. Both BOV (+21–72%) and BHV (+94–158%) increased during the maximal voluntary muscle exertions for all age levels (α < 0.05), suggesting an increased vascular recruitment to maintain oxygen homeostasis in the brain. Intriguingly, BHV was >100 folds for both resting and challenged conditions (α < 0.001) in >80% of adults aged above 50 y despite similar BOV compared with young age counterparts, indicating a huge cost of amplifying hemodynamic oscillation to maintain a stable oxygenation in the aging brain. Since vascular endothelial cells are short-lived, our results implicate a hemodynamic compensation to emergence of daily deficits in replacing senescent endothelial cells after age 50 y.

## INTRODUCTION

Brain has very high metabolic activity and consumes a disproportionate amount of energy relative to its size in a human body [1]. While size and function of the brain decline with age [2], the age-dependent increases in brain metabolic activity implies an increasing compensatory effort to sustain normal brain function [3]. The underlying nature of this compensatory mechanism is not fully understood.

A simple method to assess brain health is examining maximal voluntary handgrip strength [4] which represents the amount of muscle fibers recruited by a central command from cerebral cortex [5]. The handgrip task using a dynamometer allows measurements of the static force produced by the hand with good intra- and inter-tester reliabilities. During a voluntary muscle contraction, increasing oxygen supply to the brain requires immediate mobilization of vascular system [6–8]. Optimal blood distribution requires fast alternations of vasorelaxation and vasoconstriction in local capillaries of the brain to match the metabolic need. Therefore, each neuron receives oxygen-carrying blood for a transient moment before depletion of anaerobic fuel stored in local brain tissues. The amplitude of this hemodynamic fluctuation mirrors the vascular effort in swapping oxygen among cells within activated region of the brain [7, 8].

The real-time changes of blood distribution in the prefrontal brain can be monitored by a non-invasive near-infrared spectroscopy (NIRS) using a single detector probe [9]. During the NIRS measurement, blood distribution in the capillaries of the tissue can be optically detected by tracing mobile hemoglobin concentration (oxy- and deoxy-hemoglobin) at high frequency [10], whilst the oxygenation levels (% oxygen saturation) can be calculated as the oxyhemoglobin-to-total hemoglobin ratio during the same measuring period [11]. A technical shortfall of the NIRS measurement is different baseline values in the tissue of interest associated with site-to-site variations in cytochrome levels [12], which limits inter-tissue and inter-individual comparison. This problem can be circumvented by transforming the optical data into a variability scale (i.e., standard deviation, variance, range, interquartile range… etc.). Such variables allow quantifying the magnitude of hemodynamic oscillation to maintain oxygenation stability. In this study, the novel dimensionless indicatives, namely brain oxygenation variability (BOV) and brain hemodynamic variability (BHV), were developed by transforming a series of real-time data into standard deviation values to reveal the effort of vascular system to maintain oxygen homeostasis in the human brain. The algorithm takes the square root of the mean of the squared deviations of the optical values subtracted from their mean value. Therefore, both indicators allow quantitating fluctuation amplitude of a set of mobile hemoglobin and oxygenation values for different participants regardless their baseline level. Low BOV represents high stability towards a set point of oxygen homeostasis in the brain with a sufficient control of the vascular system. BHV reflects the magnitude of vascular oscillation to maintain stable oxygenation in the brain.

Vascular endothelial function is known to decline with age [13–15]. To address the problem of discrepancy between decreased vascular endothelial function and increased brain metabolic activity during aging [3], we examined the magnitudes of fluctuation in total hemoglobin (BHV) and % oxygen saturation (BOV) at rest and during a maximal voluntary handgrip test for 834 adults across a wide age range (20 to 88 y).

## RESULTS

**Figure 1:**
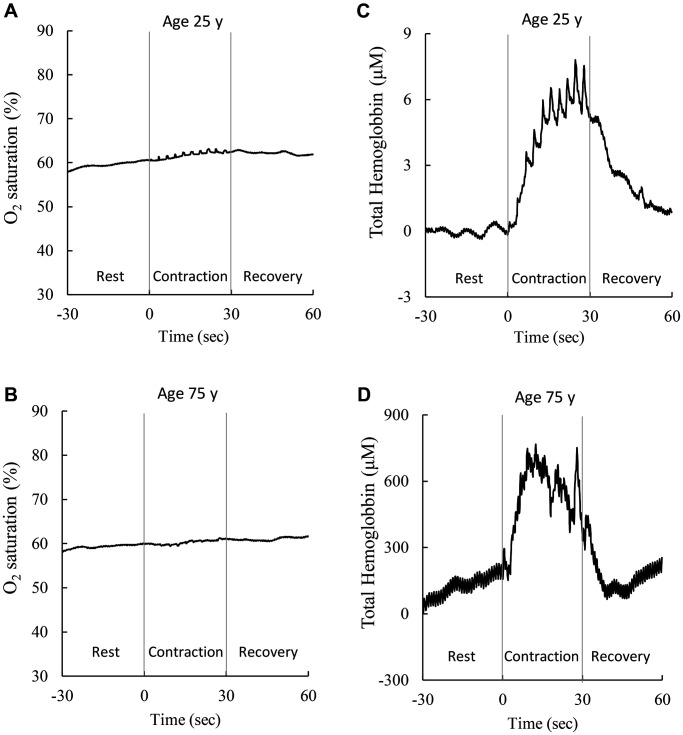
**Representative cerebral hemodynamic response during a maximal handgrip strength test.** BOV (amplitude or SD of oxyhemoglobin-to-total hemoglobin ratio) increases mildly during a 10-repeated maximal voluntary muscle exertion in adults aged 25 y (**A**) and 75 y (**B**). BHV (amplitude or SD of total hemoglobin) elevated substantially during the maximal contraction task in adults aged 25 y (**C**) and 75 y (**D**). To maintain stable oxygenation (**A**, **B**), the adult at age 75 y shows much vigorous cerebral hemodynamic response (scale increases ~100 folds) than the young counterpart at age 25 y, suggesting a huge compensatory cost of vascular control (vasoconstriction/ vasorelaxation) in the brain during muscle exertions. Abbreviations: SD: standard deviation of the 300 optical data in 30 sec; BOV: brain oxygenation variability; BHV: brain hemodynamic variability.

Sex stratification and anthropometrical characteristics of the participants are shown in Table 1. Resting BOV values were consistently low and similar for participants across the entire age range (age 20–88 y). While no difference in median BOV was found before and after age 50 y, median BHV was substantially greater in participants aged >50 y than those younger age counterparts for each quartile. Figure 1 shows examples of cerebral hemodynamic response during maximal handgrip muscle contractions (10 repetitions in 30 sec) of men aged 25 y and 75 y, respectively. The amplitude of fluctuations in cerebral oxygenation (oxyhemoglobin-to-total hemoglobin ratio, BOV) against the 30-sec muscle contraction was small (A, B), whereas cerebral hemoglobin fluctuation was increased substantially above the rest phase (C, D) for both age levels. While the response patterns against muscle exertion are similar for both ages, the scale of such changes in cerebral hemoglobin during a maximal handgrip muscle strength test was substantially greater (~100 folds) for the adult aged 75 y (D) than 25 y (C), indicating a greater vascular struggle in the older adult to maintain a stable oxygenation in the brain.

**Table 1:** Participant characteristics.

**Age (*N* = 834)**	**20–50 y**	**51–88 y**	***p***
Sex	M/F: 245/235	M/F: 141/213	–
Weight (kg)	67 (38–132)	62 (39–113)	<0.001
Height (cm)	168 (147–195)	161 (143–187)	<0.001
BMI	23.8 (16.0–48.9)	23.8 (15.0–37.6)	NS
Lean mass (kg)	46.7 (24.6–83.4)	39.8 (24.9–72.5)	<0.001
Fat mass (kg)	17.7 (4.7–64.6)	19.3 (6.0–41.4)	0.001
Bone mass (g)	2.61 (1.38–4.21)	2.14 (1.18–3.89)	<0.001
BMD (g/cm^2^)	1.23 (0.80–1.61)	1.08 (0.69–1.54)	<0.001
BOV-Q1	0.26 (0.10–0.33)	0.23 (0.10–0.34)	NS
BOV-Q2	0.42 (0.33–0.51)	0.42 (0.34–0.51)	NS
BOV-Q3	0.66 (0.51–0.86)	0.59 (0.51–0.78)	NS
BOV-Q4	1.30 (0.86–11.64)	1.02 (0.79–3.26)	NS
BHV-Q1	0.30 (0.01–0.42)	0.76 (0.01–42)	<0.001
BHV-Q2	0.56 (0.43–0.72)	66 (43–96)	<0.001
BHV-Q3	0.90 (0.73–1.24)	120 (96–173)	<0.001
BHV-Q4	1.91 (1.25–186)	260 (174–683)	<0.001

Data is expressed as median (minimum-maximum). Abbreviations: BMI: body mass index; BMD: bone mineral density; BOV: brain oxygenation variability at rest; BHV: brain hemodynamic variability at rest. Lower resting values of BOV and BHV represent higher hemodynamic stability. Q1–Q4 represents each quartile of the age range investigated.

**Figure 2:**
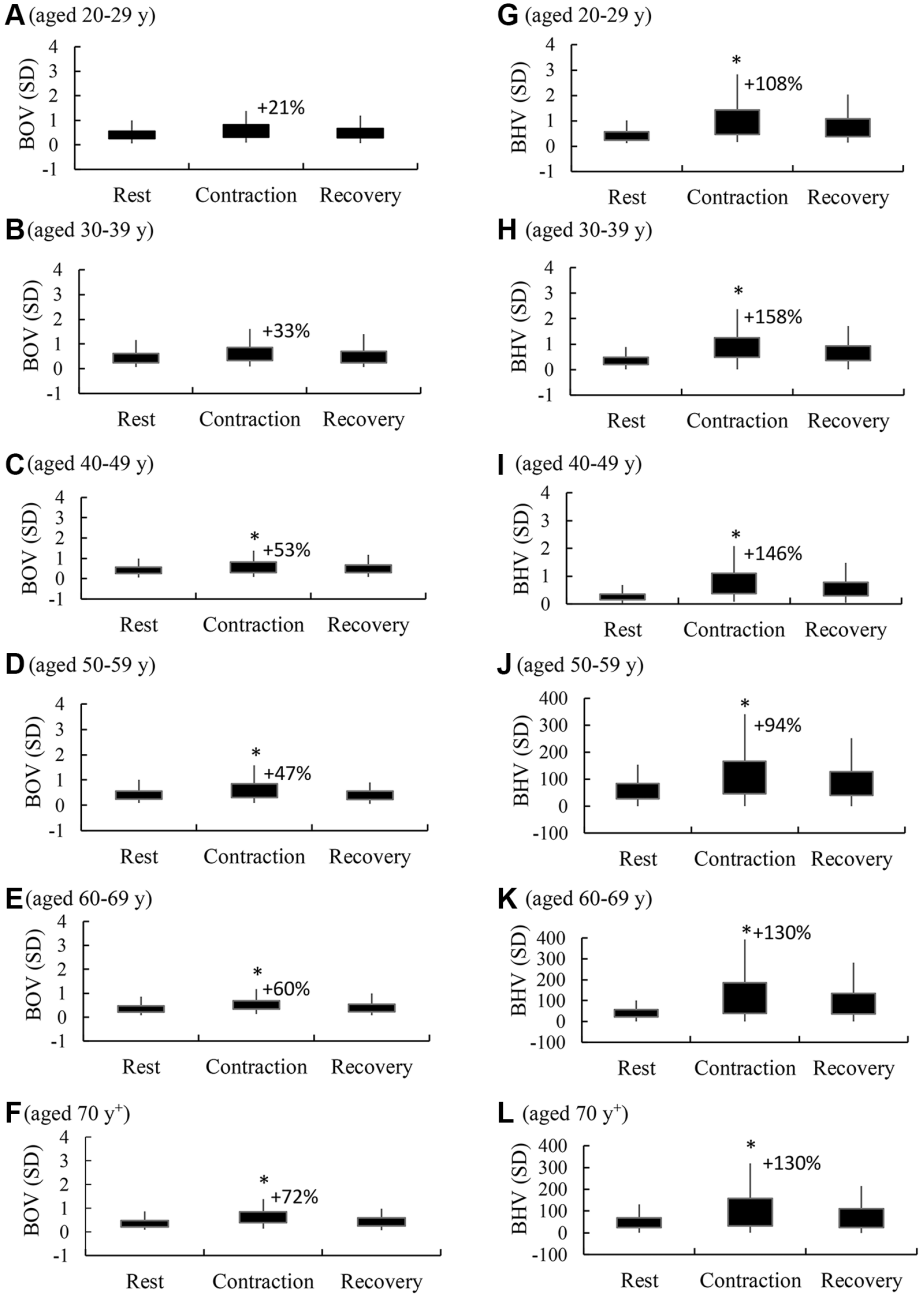
**Age breakdown of brain hemodynamic response against a maximal handgrip strength test.** BOV (standard deviation or SD of oxyhemoglobin-to-total hemoglobin ratio) (**A**–**F**) reflects the magnitude of brain oxygenation fluctuation during a 30-sec rest phase, a 30-sec contraction phase (10 repetitions), and a 30-sec recovery phase. BHV (standard deviation or SD of tissue hemoglobin values) (**G**–**L**) represents the magnitude of brain hemodynamic struggle for maintaining oxygenation stability during the 3 corresponding phases. ^*^α < 0.05 compared against the rest phase. Abbreviations: SD: standard deviation of the 300 optical data in 30 sec; BOV: brain oxygenation variability; BHV: brain hemodynamic variability.

Age breakdown of BOV and BHV responses against maximal handgrip muscle contractions are presented separately in Figure 2. A significant main effect of exercise response for both BOV and BHV was consistently observed (rest vs. contraction, α < 0.01). This acute response of BOV and BHV returned quickly during the 30-s recovery period (contraction vs. recovery, α < 0.05). Small increases in BOV above resting levels (+21% and +33%) during a maximal handgrip muscle strength test were observed in the young adults aged <40 y (A, B) (main effect of time, α < 0.05). From age 40–88 y, the muscle contraction-induced increases in BOV above rest were doubled (C, D, E, F) (main effect of time, α < 0.05). The muscle contraction-induced increases in BHV were mostly >100% above the rest phase at all age levels (G, H, I, J, K, L). Despite a similar pattern across all ages, the scale of the BHV changes for adults aged after 50 y was >100 folds of their young age counterparts (main effect of time, α < 0.001).

**Figure 3:**
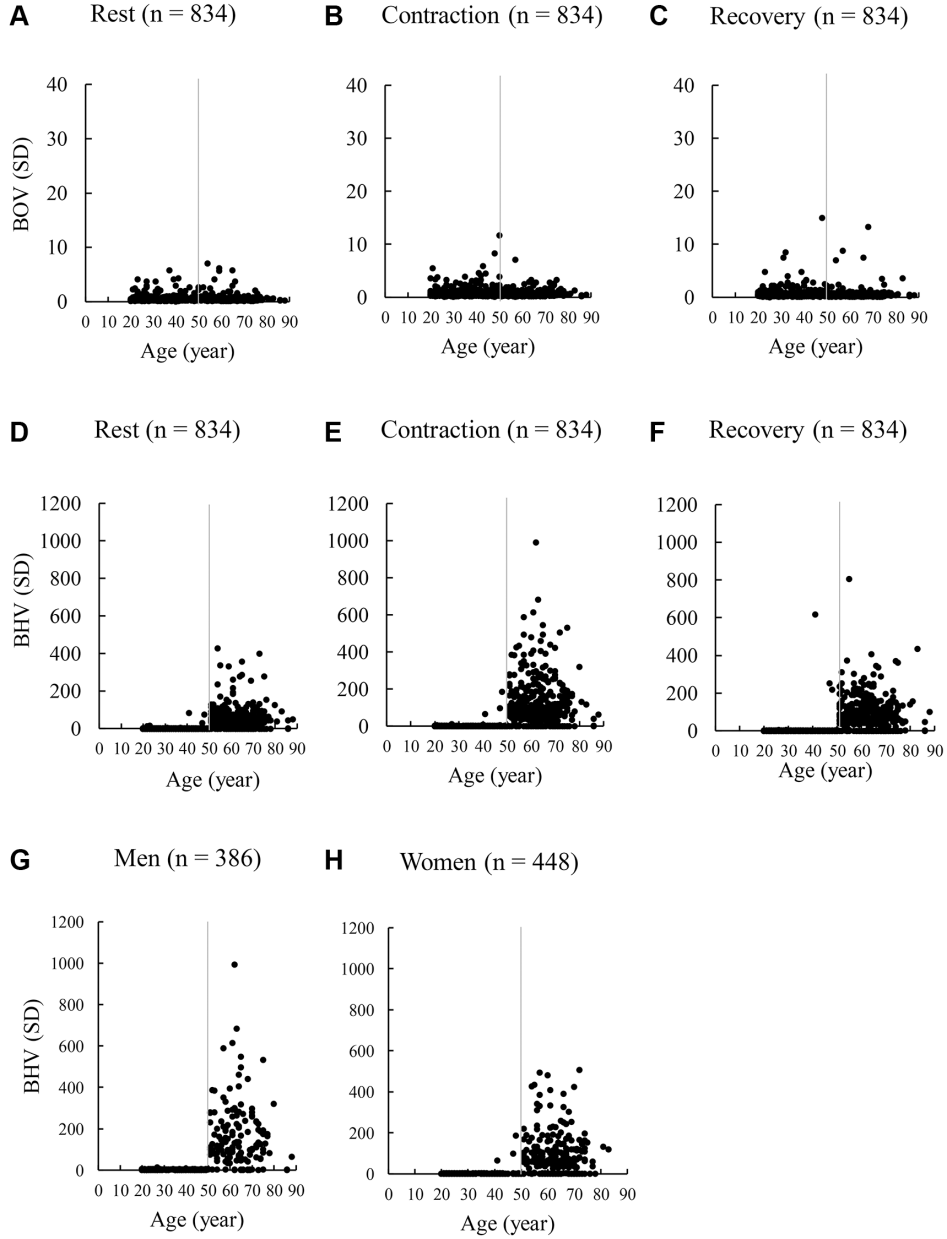
**Scatter plots of BOV and BHV during a maximal handgrip strength test of 834 adults aged 20–88 y.** The 30-sec handgrip test includes a 10-repeated voluntary maximal exertion on a dynamometer with 3-sec rest intervals. BOV values (**A**–**C**) are calculated as standard deviation of oxygen saturation values during the 3 phases: a 30-sec rest (300 values), a 30-sec contraction (300 values), and a 30-sec recovery (300 values). Similarly, BHV values (**D**–**F**) are calculated as SD of total hemoglobin values during the 3 phases (rest, contraction, recovery). A sharp elevation of BHV after age 50 y was apparent for >80% of adults, similar for men (**G**) and women (**H**) during voluntary muscle exertions at maximal effort. Abbreviations: SD: standard deviation of the 300 optical data in 30 sec; BOV: brain oxygenation variability; BHV: brain hemodynamic variability.

Figure 3 shows scatter plots of BOV and BHV values of all participants (*n* = 834) from age 20 to 88 y. This result indicates an apparently low BOV values during rest (A), contraction (B), and recovery (C) (stable oxygenation levels) compared with BHV for participants aged 20–88 y, suggesting a preservation of high stability in brain oxygenation across a wide age range. BHV values in the individuals aged above 50 y (D, E, F) were much higher than their young age counterparts (aged 20–50 y). A sharp cutoff point after age 50 for the age-dependent increases in BHV was very prominent for both men and women (G, H). Therefore, potential bias is unlikely. The ratio of population escaped from normal range (95% CI of 20–29 y) of BHV values does not appear to increase until age 50 y. In particular, the percentage of the population exceeding the upper ceiling of normal range (95% CI) of their young age counterparts are 11% (20–29 y), 11% (30–39 y), and 7% (40–49 y), 85% (50–59 y), 81% (60–69 y), and 80% (70+ y) (α < 0.001). The sharp cut-off point of BHV after age 50 y is also similar at rest and recovery phases (D, F).

**Figure 4:**
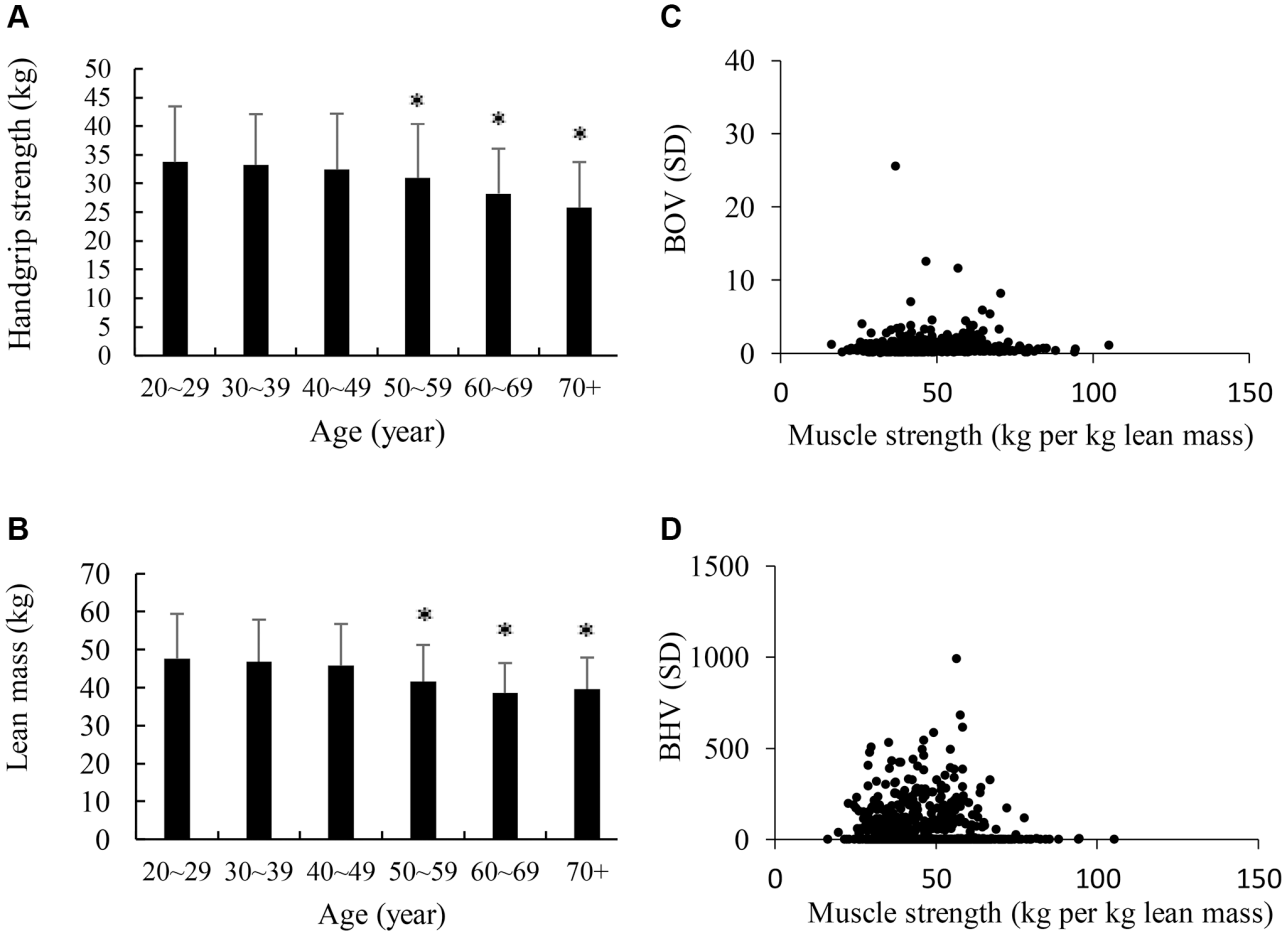
**Maximal voluntary muscle strength and brain hemodynamic fluctuation at higher ages.** Both maximum handgrip strength (**A**) and lean body mass (**B**) of adults aged >50 y were moderately lower than their young age counterparts. BOV was low (<10) across a wide range of relative muscle strength (kg/lean mass in kg) among participants (**C**). Participants with high voluntary muscle strength shows mostly low BHV during maximal handgrip contraction (**D**), suggesting a low vascular compensating effort to limit oxygen fluctuation within a small range. ^*^α < 0.05 compared against the young control adults (20–29 y). Abbreviations: SD: standard deviation of the 300 optical data in 30 sec; BOV: brain oxygenation variability; BHV: brain hemodynamic variability.

Figure 4 presents the average values of maximal handgrip muscle strength and lean body mass of participants from age 20 to 88 y. Adults aged >50 y had slightly lower maximal handgrip muscle strength (A) and lean body mass (B) compared with those at younger aged 20–29 y (α < 0.05). The scatter plot shows a non-linear relationship between BOV and muscle strength (kg per kg body weight in percentage) (C). Adults with the highest muscle strength (>75 kg per kg body weight) showed very low incidence of both high BOV and high BHV compared with those with low muscle strength counterparts (D).

## DISCUSSION

### Major findings

Vascular endothelial function is known to decrease with age [13, 14]. Intriguingly, metabolic activity of the prefrontal brain elevates with age [16]. This discrepancy implicates an age-dependent compensatory effort to sustain oxygenation in the brain. Oxygen delivery to match metabolic demand among cells relies on fast vasorelaxation and vasoconstriction by flashing oxygenated blood in tissues. Therefore, we developed BOV and BHV to indicate the magnitude of real-time fluctuation of oxygenation and blood distribution in the prefrontal brain. In this study, we validated that BHV and BOV as an indicator or brain vascular struggle using maximal voluntary handgrip exertions. The major findings of the study are as follows: 1) BHV increased substantially while BOV increase to a lesser extent during an episode of muscle contractions at a maximal effort; 2) BHV at rest and during maximal handgrip exertions increased >100 folds for most of adults age >50 y, suggesting an amplified vasorelaxation and vasoconstriction effort to maintain normal brain oxygenation stability (suppressed BOV); 3) Brain oxygenation stability during maximal handgrip contractions seems to be well-preserved and remained similar from age 20 to 88 y. These results provide a mechanistic explanation of increased metabolic activity of the prefrontal brain [16] as a physiological compensation during vascular aging [13, 14], and indicates that the vascular aging accelerates after age 50 y. A sharp increase in BHV indicates a huge hemodynamic compensation against a cliff-like vascular deterioration in the aging brain, in contrast with our conventional view that aging is a gradually occurring process [17].

Maximal voluntary handgrip strength is a well-established indicative of brain health [4]. In this study, we provide further evidence which indicates that the vascular control in the brain is directly involved with mobilization of muscles by central commands. In the study, we selected 10 attempts at maximal exertion efforts based on our preliminary test of brain hemoglobin elevation in which plateau cannot be achieved by 3 attempts for most of participants. Most of previous studies used 3 attempts to indicate the maximal voluntary effort during handgrip contractions [18]. A very high correlation between average maximal muscle strength and the peak muscle strength of 10 attempts in our study suggests no difference of using both methods to obtain maximal handgrip strength.

### The compensatory mechanism against brain vascular aging

In the present study, sharply increased oscillations in cerebral hemoglobin (reflected by increased BHV) for most of adults > age 50 y may be a compensation to the loss of anaerobic capacity and/or deteriorated endothelial function. It is worthy to note that the age-dependent increases in BHV after age 50 y is also evident at rest. A recent study from multi-tracer PET brain imaging suggests a metabolic shift from mixture of nonoxidative and oxidative use of glucose towards predominantly oxidative metabolism during advancing age [19]. Glycolysis decreases to nearly zero at the age of 60 y [19]. These results partly explain an increased oxygen extraction in the aging brain [20]. Age-dependent deterioration in endothelial function is known as a major contributor for functional decline in older adults [13]. Endothelial cell senescence is expected to compromise efficiency of oxygen and nutrient transport to the brain. To adapt with this age-dependent deterioration, a hemodynamic compensation would be required to sustain cell survival in the brain.

### The cliff-like vascular deterioration

A sharp increase in BHV after age 50 implicit a fast deterioration occurring in the vascular system within a year period. The underlying mechanism accounted for the fast deterioration is far from clear. We speculate that this rapid decline is associated with an emergence of daily deficit in endothelial cell renewal. Endothelial cells in capillary of mammalian tissues have a short lifespan around 2 weeks [21]. Therefore, a 100% daily replacement of endothelial cells is required to maintain youth and functional vascular endothelium for oxygen delivery to the surrounding neurons. Assuming the daily renewal rate of endothelial cells decreases from 100% to 98%, unreplaced senescent endothelial cells can rapidly accumulate and occupy to 87% in the entire endothelial lumen of capillary in 100 days, leading to a cliff-like vascular deterioration. The observed increases in BHV with a relatively stable BOV suggests an enormous vascular effort to shuffle more blood in and out of the brain tissue for oxygenation. Endothelial progenitor cells are produced from bone marrow to replace senescent endothelial cells in blood vessels [22]. We have previously reported an age-dependent decrease in bone to body mass ratio during natural growth in middle-age. Thus, the development of imbalance between the bone marrow cell production organ and an expanding body mass may explain the emerged deficit in endothelial cell replacement [23]. Furthermore, loss of sex hormones at age 50 y in men [24] and women [25] may partly contribute to the corresponding vascular deterioration reflected by increased BHV. Anabolic hormones like sex hormone and insulin play a key role in basal bone marrow cell proliferation [23, 26] to sustain vascular function [27].

### Limitation of the study

In this study, we did not find age-dependent changes in BHV across the age from 20 to 50 y. Following a sharp increase in BHV after age 50, no further age-dependent changes in BHV was observed thereafter. We could not preclude the possibility that there may be a ceiling for survival in the vascular compensation by amplifying BHV for oxygen homeostasis after age 50 y. When BHV is elevated to an extent of compensation failure for oxygen homeostasis, the individuals can no longer survive and absent as a participant in the study. A longitudinal observation for a wide population around age 50 y would be needed to clarify this possibility.

## CONCLUSION

The present study validates BOV and BHV as novel indicatives to reflect the ability to maintain oxygenation and blood distribution, respectively, in the brain. Low levels of BOV and BHV represent better oxygenation stability with a relaxed effort of vascular controls. Maximal voluntary muscle contraction increased BHV, and to a minor extent, BOV suggesting a priority to sustain brain oxygenation within a narrow range in cost of increasing hemodynamic control. The most striking finding of the study is a sharp increase in BHV at rest and during muscle contraction for most of men and women after age 50 y, while BOV remains stably low across a wide age range. This huge compensation effort to increase BHV after age 50 y implicates the emergence of age-onset deficit in daily replacement of short-lived endothelial cells leading to accumulations of senescent/unfunctional endothelial cell population in the vasculatures within a year.

## METHODS

### Experimental approach to the study

To determine how a cerebral oxygenation stability is maintained during a maximal voluntary muscle contraction effort and whether the hemodynamic control to maintain this oxygenation stability is compromised at higher age, oxygen saturation and total hemoglobin levels were monitored at rest and exercise in 834 participants (height 164.9 ± 9.2 cm, weight 65.0 ± 14.1 kg) aged 20–88 y from the Taipei City. Exclusion criteria were inability to conduct a maximal handgrip strength test and orthopedic implantation of metal.

Participants gave their written informed consent before the test and University of Taipei Institutional Review Board approved the study protocol (IRB-2018-073). All experimental procedures were conducted in accordance with the spirit of Declaration of Helsinki.

Participants reported to the laboratory on a single occasion. They were required to abstain from strenuous exercise and alcohol consumption 48 h prior to the experimental session. For the entire duration of the experimental procedures, participants were required to remove any accessories. They were familiarized with all the experimental procedures protocol and researcher corrected the procedure, if needed. They were asked to identify their dominant arm.

### Brain hemodynamic measurement

BOV and BHV were measured using a wireless NIRS device (PortaLite, Artinis Medical System, Elst, Netherlands) before, during, and after the handgrip muscle strength test. A NIRS optical detector was placed on the left frontal region if dominant arm was right hand, vice versa. Participants were familiarized with the protocol and an experimenter corrected the procedure during a test trial. They sat in a chair at the most comfortable upright position and put hand straight on a table in the front during the entire testing procedure with a few adjustments. Participants were then informed about the 3 phases of the measurements: rest (30 sec), 10-grips (30 sec), recovery (30 sec). Since mental and physical disturbance can vary NIRS values in the brain, experimenters paid special attention to calm participants before establishing a baseline. Participants were asked to relax completely until a stable baseline was established for 1 min before recording the 30-s rest phase. The baseline is defined by minimal fluctuations in oxygenation (% oxygen saturation) and total hemoglobin without an obvious upward or downward trend for 1 min. During the 30-s rest phase, pacer was turned on 10 sec before the first grip. During contraction phase, participants started the grip at maximal effort every 3 sec (fingers always attached the device) according to the pacer. Following the handgrip test, brain hemodynamic changes continued to record for another 30 sec during the recovery phase. The entire procedure was repeated with a 3 min interval. The test-retest reliability in BHV was 0.71 (*p* < 0.01).

Data of total hemoglobin and oxygenation (% oxygen saturation) were collected at 10 Hz via bluetooth using Oxysoft software (Artinis Medical Systems, Elst, Netherlands). We transformed the real-time data of 300 values (10 Hz in 30 sec) into variability (standard deviation, SD) of total hemoglobin (BHV) and oxygenation (BOV) to reflect the amplitude of hemodynamic fluctuation. Since mobile hemoglobin values are a direct estimate of blood concentration in tissues, BHV reflects the magnitude of vascular regulation resulted from fast vasoconstriction and vasorelaxation to maintain brain oxygenation (i.e., low BOV reflects high oxygen stability).

### Handgrip muscle strength test

Maximal handgrip muscle strength was measured using a Takei Dynamometer Model T.K.K.5401 (Takei Scientific Instruments Co., Niigata, Japan). To standardize the protocol in preventing difference in maximal muscle strength due to the angle of the elbow, participants sat straight-backed in a chair at the most comfortable position with hand straight. After a practice on the dynamometer, participants completed 10 maximal voluntary exertions within 30 sec between each trial, which delineated a typical pattern of hemodynamic response. The entire procedure was repeated twice and separated by a 3 min rest interval for the purpose to determine the test-retest reliability. The peak muscle strength and mean strength of the 10 maximal exertions had nearly perfect correlation (*r* = 0.98, *p* < 0.001). Test-retest reliability (*n* = 834) of the two handgrip tests of mean maximal strength was very high (*r* = 0.94, *p* < 0.001). Therefore, mean maximal strength was used to calculate statistical significance.

### Lean body mass

On the day of the test, the participants were asked to remove metal accessories. Lean body mass was assessed using dual-energy X-ray absorptiometry (DXA). After assessments of weight and height, lean body mass of all participants was measured by a whole-body DXA scanner (Lunar iDXA; GE Medical Systems, Madison, WI, USA) using Encore software V.13.60.033 (Encore, Madison, WI, USA) in normal mode.

### Statistical analysis

Shapiro-Wilk test was used to test whether all variables were normally distributed. Homogeneity of variances was confirmed by Levene test. Since the data for absolute total hemoglobin and tissue oxygenation levels (oxyhemoglobin-to-total hemoglobin ratio) are not normally distributed, BOV and BHV are expressed as median (95% confidence interval or CI). To indicate the percentage of the population exceeding the upper ceiling of normal range for BHV for each age levels, we considered extreme values as 95% CI above the young age group (20–29 y) and was tested by Chi square. Friedman test was used and when a significant F ratio was found, Mann–Whitney *U* test was used for post hoc analysis. A level of significance was set at an α = 0.05 for all variables, and values are expressed as median (95% CI). Mean value of 10 maximal handgrip strength was used for statistical analysis. Pearson correlation was calculated for the variables between the first handgrip test and the second handgrip test. SPSS 25.0 was used for statistical analysis.
